# Toward accurate quantitative photoacoustic imaging: learning vascular blood oxygen saturation in three dimensions

**DOI:** 10.1117/1.JBO.25.8.085003

**Published:** 2020-08-24

**Authors:** Ciaran Bench, Andreas Hauptmann, Ben Cox

**Affiliations:** a University College London, Department of Medical Physics and Biomedical Engineering, Gower Street, London, United Kingdom; b University of Oulu, Research Unit of Mathematical Sciences, Oulu, Finland; c University College London, Department of Computer Science, Gower Street, London, United Kingdom

**Keywords:** photoacoustics, deep learning, oxygen saturation, sO_2_, machine learning, quantitative photoacoustics

## Abstract

**Significance:** Two-dimensional (2-D) fully convolutional neural networks have been shown capable of producing maps of ${\mathrm{sO}}_{2}$ from 2-D simulated images of simple tissue models. However, their potential to produce accurate estimates *in vivo* is uncertain as they are limited by the 2-D nature of the training data when the problem is inherently three-dimensional (3-D), and they have not been tested with realistic images.

**Aim:** To demonstrate the capability of deep neural networks to process whole 3-D images and output 3-D maps of vascular ${\mathrm{sO}}_{2}$ from realistic tissue models/images.

**Approach:** Two separate fully convolutional neural networks were trained to produce 3-D maps of vascular blood oxygen saturation and vessel positions from multiwavelength simulated images of tissue models.

**Results:** The mean of the absolute difference between the true mean vessel ${\mathrm{sO}}_{2}$ and the network output for 40 examples was 4.4% and the standard deviation was 4.5%.

**Conclusions:** 3-D fully convolutional networks were shown capable of producing accurate ${\mathrm{sO}}_{2}$ maps using the full extent of spatial information contained within 3-D images generated under conditions mimicking real imaging scenarios. We demonstrate that networks can cope with some of the confounding effects present in real images such as limited-view artifacts and have the potential to produce accurate estimates *in vivo*.

## 1 Introduction

Blood oxygen saturation (${\mathrm{sO}}_{2}$) is an important physiological indicator of tissue function and pathology. Often, the distribution of oxygen saturation values within a tissue is of clinical interest, and therefore, there is a demand for an imaging modality that can provide high-resolution images of ${\mathrm{sO}}_{2}$. For example, there is a known link between poor oxygenation in solid tumor cores and their resistance to chemotherapies, thus images of tumor blood oxygen saturation could be used to help stage cancers and monitor tumor therapies.^1^^,^^2^ Some imaging modalities have been shown capable of providing limited information about or related to ${\mathrm{sO}}_{2}$ in tissue. Blood oxygenation level-dependent magnetic resonance imaging, which is sensitive to changes in both blood volume and venous deoxyhemoglobin concentration, can be used to image brain activity, but cannot respond to changes in oxygen saturation.^3^ Purely optical techniques, such as near-infrared spectroscopy and diffuse optical tomography, can be used to generate images of oxygen saturation.^4^^,^^5^ However, because of high optical scattering in tissue, these modalities can only generate images with low spatial resolution beyond superficial depths.

Photoacoustic (PA) imaging is a hybrid modality that can be used to generate high-resolution images of vessels and tissue at greater imaging depths than purely optical modalities.^6^ PA image contrast depends on the optical absorption of the sample, so images of well-perfused tissues and vessels can, in principle, be used to generate images of ${\mathrm{sO}}_{2}$ with high specificity. However, unlike strictly optical techniques, information about the contrast in PA images is carried by acoustic waves that can propagate from deep within a tissue to its surface undergoing little scattering. In the ideal case of a perfect acoustic reconstruction, the amplitude of a voxel in a PA image can be described as

$$p_{0}(x,\lambda )=\mu_{a}(x,\lambda )\Gamma (x)\Phi (x,\lambda ;\mu_{a},\mu_{s},g),$$ (1)

where x is the voxel’s location within the sample, λ is the optical wavelength, $\mu_{a}$ is the optical absorption coefficient, $\mu_{s}$ is the scattering coefficient, *g* is the optical anisotropy factor, Γ is the PA efficiency (assumed here to be wavelength independent), and Φ is the light fluence. Images of ${\mathrm{sO}}_{2}$ may only be recovered if the sample’s absorption coefficients [or at least the absorption coefficient scaled by some wavelength-independent constant, such as $\mu_{a}(x,\lambda )\Gamma (x)$] can be extracted from each image. In the hypothetical case where the sample’s fluence distribution is constant with wavelength, a set of PA images acquired at multiple wavelengths automatically satisfies this requirement. However, because the optical properties of common tissue constituents are wavelength dependent, this condition is never met in *in vivo* imaging scenarios.^7^ In general, knowledge of the fluence distribution throughout the sample at each excitation wavelength is required to accurately image ${\mathrm{sO}}_{2}$.^8^ If an accurate fluence estimate is available, then an image of the sample’s relative optical absorption coefficient at a particular wavelength can be obtained by performing a voxelwise division of the image by the corresponding fluence distribution, as described by

$$\frac{p_{0}(x,\lambda )}{\Phi (x,\lambda )}=\mu_{a}(x,\lambda )\Gamma (x).$$ (2)

In some cases, it might be possible to measure an estimate of the fluence using an adjunct modality,^9^ but more commonly, attempts have been made to model the fluence. However, because the optical properties of a tissue sample are usually not known before imaging (the only reason the fluence is estimated at all is so that unknown information about the sample’s optical absorption coefficient can be recovered from the image data), it is difficult to model the fluence distribution. A variety of techniques have been developed to recover tissue absorption coefficients from PA images without total prior knowledge of the tissue’s optical properties. Progress toward solving this problem can be summarized into three key phases. In the first phase, one-dimensional analytical fluence models were used to estimate the fluence by taking advantage of assumed prior knowledge of some of the sample’s optical properties, or by extracting the optical properties of the most superficial layers from image data.^10^^–^^15^ In the latter case, the effective attenuation coefficient of the most superficial tissue layer (assumed to be optically homogeneous) is usually estimated by fitting an exponential curve to the decay profile of the image amplitude above the region of interest (e.g., a blood vessel).

In the next phase, sample optical properties were recovered using iterative error minimization approaches.^16^^–^^19^ With these techniques, knowledge of the underlying physics is used to formulate a model of image generation. The set of model parameters (which might include the concentrations of deoxyhemoglobin and oxyhemoglobin in each voxel) that minimizes the error between the images generated by the model and the experimentally acquired images are treated as estimates of the same parameters in the real images. This technique is only effective when the model of image generation is able to generate a set of simulated images very similar to the real set of images when the correct values for the chromophore concentrations are estimated. This is only possible when the image generation model is able to accurately model image acquisition in the real system. In practice, accurate models of image generation are challenging to formulate as not all aspects of the data acquisition pathway are fully characterized. Therefore, this technique has not yet been shown to be a consistently accurate method for imaging ${\mathrm{sO}}_{2}$ in tissue. Both iterative error-minimization and analytical techniques may require significant *a priori* knowledge of sample properties, such as all the different constituent chromophore types. This information is not always available when imaging tissues *in vivo*, and thus this requirement further reduces their viability as techniques for estimating ${\mathrm{sO}}_{2}$ in realistic imaging scenarios. The recent emergence of a third phase has introduced data-driven approaches for solving the problem.^20^^–^^28^ With these approaches, generic models are trained to output images of ${\mathrm{sO}}_{2}$ or optical properties by processing a set of examples.^29^ These data-driven models find solutions without significant *a priori* knowledge of sample properties and do not require the formulation of an image generation model using assumed prior knowledge of all the aspects related to image acquisition. Techniques based on data-driven models, such as deep learning, have been used to estimate ${\mathrm{sO}}_{2}$ from two-dimensional (2-D) PA images of simulated phantoms and tissue models.^20^^–^^22^^,^^24^^,^^25^^,^^28^ Fully connected feedforward neural networks have been trained to estimate the ${\mathrm{sO}}_{2}$ in individual image pixels given their PA amplitude at multiple wavelengths.^20^ Because the fluence depends on the three-dimensional (3-D) distribution of absorbers and scatters, a pixelwise approach does not use all of the information available in an image. Encoder–decoder type networks, capable of utilizing spatial as well as spectral information, have been trained to process whole multiwavelength 2-D images of 2-D tissue models,^22^^,^^24^^,^^25^^,^^28^ or 2-D images sliced from more realistic 3-D tissue models featuring reconstruction artifacts,^21^ and output a corresponding 2-D image of the ${\mathrm{sO}}_{2}$/optical absorption coefficient distribution. Although 2-D convolutional neural networks can take advantage of spatial information to improve estimates of ${\mathrm{sO}}_{2}$, networks trained on 2-D images sliced from 3-D images are missing information contained in other image slices that might improve their ability to learn a fluence correction. 3-D networks are often better at learning tasks requiring 3-D context.^30^^–^^32^ Therefore, it is important to show that networks can take advantage of all four dimensions of information from a multiwavelength PA image dataset to estimate ${\mathrm{sO}}_{2}$. In addition to supervised learning, an unsupervised learning approach has been used to identify regions containing specific chromophores (such as oxyhemoglobin and deoxyhemoglobin) in 2-D simulated images.^23^ The technique has not yet been used to estimate ${\mathrm{sO}}_{2}$ and has only been tested on a single simulated phantom lacking a complex distribution of absorbers and scatterers that would normally be found in *in vivo* imaging scenarios.

As we aim toward developing a technique for estimating 3-D ${\mathrm{sO}}_{2}$ distributions from *in vivo* image data, a more robust demonstration of a data-driven technique’s ability to acquire accurate ${\mathrm{sO}}_{2}$ estimates by processing whole 3-D images of realistic tissue models is desired. We trained two encoder–decoder type networks with skip connections to (1) output a 3-D image of vascular ${\mathrm{sO}}_{2}$ and (2) output an image of vessel locations from multiwavelength (784, 796, 808, and 820 nm) images of realistic vascular architectures immersed in three-layer skin models, featuring noise and reconstruction artifacts.

Ideally, networks would be trained on *in vivo* data to demonstrate their ability to cope with all the confounding effects present in real images. However, because there is no reliable technique to acquire ground truth ${\mathrm{sO}}_{2}$ data *in vivo*, generating such a dataset is very difficult. Blood flow phantoms can be used to generate images with accompanying information about the ground truth ${\mathrm{sO}}_{2}$.^33^^,^^34^ However, these phantoms are usually much simpler than real tissue (e.g., optically homogeneous tissue backgrounds, tube-shaped vessels) and thus are not ideal for assessing whether networks can produce accurate estimates in more realistic cases. To overcome this, simulated images of realistic tissue models with known ground truths were used instead. The drawback with this approach is that simulations cannot capture every aspect of a real measurement, e.g., the noise and sensor characteristics may not be well known. Nevertheless, using training data that has been simulated in 3-D with limited-view artifacts, a gold-standard light model, realistic optical properties, and noise levels provides a good indication of the network’s ability to cope with measured data. Furthermore, given the difficulty of obtaining measured data with a ground truth, pretraining with realistic simulation data could be a very useful step prior to transfer training with a limited amount of measured data. Details about how the simulated images were generated are described in Sec. 2. Section 3 describes the network architecture and details about the training process. Section 4 describes the results.

## 2 Generating Simulated Images

Ideally, a network trained to estimate ${\mathrm{sO}}_{2}$ from *in vivo* images would be capable of generating accurate estimates from a wide range of tissue samples with varying optical properties and distributions of vessels. In addition, the network should be able to do this despite the presence of reconstruction artifacts and noise. This section describes each step involved in the generation of the simulated images used in this study.

### 2.1 Tissue Models

**Fig. 1:**
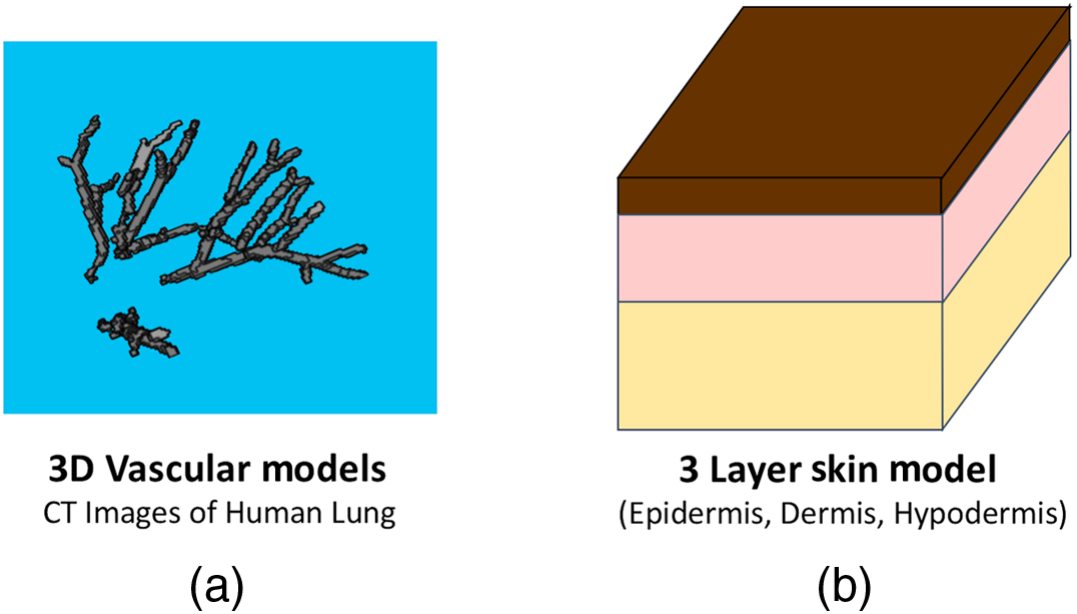
(a) Example of a 3-D vessel model (acquired from CT images of human lungs) used to construct 3-D tissue models. (b) Schematic of three-layer skin model used to construct tissue models.

A set of several hundred tissue models, each featuring a unique vascular architecture and distribution of optical properties, were generated by immersing 3-D vessel models acquired from computed tomography (CT) images of human lung vessels into 3-D, three-layer skin models (some examples are shown in Fig. 1).^35^^,^^36^ Each skin model contained three skin layers (an epidermis, dermis, and hypodermis). The thickness of each skin layer (epidermis: 0.1 to 0.3 mm, dermis: 1.3 mm to 2.9 mm, hypodermis: 0.8 mm to 2.6 mm), and the optical absorption properties of the epidermis and dermis layers were varied for each tissue model. A unique tissue model was generated for each vascular model. The equations used to calculate the optical properties of each skin layer and the vessels at each excitation wavelength (784, 796, 808, and 820 nm) are presented in Table 1 in Appendix B. These wavelengths were chosen as they fell within the near-infrared (NIR), and data were available for all skin layers at these wavelengths. The absorption properties of the epidermis layer of each tissue model were determined by choosing a random value for the melanosome volume fraction that was within expected the physiological range. The absorption properties of the dermis layer were determined by choosing random values for the blood volume fraction and dermis blood ${\mathrm{sO}}_{2}$ within the expected physiological range. For each tissue model, each independent vascular body was randomly assigned one of three randomly generated ${\mathrm{sO}}_{2}$ values between 0% and 100%. The PA efficiency throughout the tissue was set to one with no loss of generality.

### 2.2 Fluence Simulations

The fluence in each tissue model at each excitation wavelength was simulated with MCXLAB, a MATLAB^®^ package that implements a Monte Carlo (MC) model of light transport (considered the gold standard for estimating the fluence distribution in tissue models).^37^ Fluence simulations were run with $10^{9}$ photons, the maximum number of photons that could be used to generate 1024 sets of images in ∼1 week using a single NVIDIA Titan X Maxwell graphics processing unit (GPU) with 3072 CUDA cores and 12 GB of memory. A large number of photons were used in order to reduce the MC variance to the point where it no longer contributed significantly to the noise in the simulated data. Noise was subsequently added in a systematic way to the simulated time series, as described in Sec. 2.3.

MC simulations were run with voxel sidelengths of 0.1 mm, and simulation volumes with dimensions of 40×120×120  voxels. Tissue models were assigned depths of 4 mm as this is the approximate depth limit for clear visualisation of vessels *in vivo* with the high-resolution 3-D scanner reported in Refs. 38 and 39. The fluence was calculated from the flux output from MCXLAB by integrating over time using timesteps of 0.01 ns for a total of 1 ns, which was sufficient to capture the contributions from the vast majority of the scattered photons. A truncated Gaussian beam with a waist radius of 140 voxels, with its center placed on the center of the top layer of epidermis tissue, was used as the excitation source for this simulation. Photons exiting the domain were terminated. The fluence simulations were not scaled by any real unit of energy, as images were normalized before inserting them into the network. Each fluence distribution was multiplied pixelwise by an image of the tissue model’s corresponding optical absorption coefficients to produce images of the initial pressure distribution at each excitation wavelength.

### 2.3 Acoustic Propagation and Image Reconstruction

Simulations of the acoustic propagation of the initial pressure distributions from each tissue model, the detection of the corresponding acoustic pressure time series at the tissue surface by a detector with a planar geometry, and the time reversal reconstruction of the initial pressure distributions from these times series were executed in k-Wave.^40^ Simulations were designed with a grid spacing of 0.1 mm, dimensions of 40×120×120  voxels, and a perfectly matched layer of 10 voxels surrounding the simulation environment. Each tissue model was assigned a homogeneous sound speed of 1500  m/s. 2-D planar sensor arrays are often used to image tissue *in vivo*, as it is a convenient geometry for accessing various regions on the body.^39^^,^^41^^,^^42^ A sensor array with a planar geometry was used in this study to mimic conditions expected in real imaging scenarios. A 2-D planar sensor mask covering the top plane of the tissue model was used to acquire the time series data. Because of its limited-view geometry, the sensor array will detect less pressure data emitted from deeper within the tissue, as these regions will subtend a smaller angle with the sensor. As a result, the reconstruction will have limited-view artifacts, which will become more pronounced with depth.^43^

To avoid the large grid dimensions that would be required to capture the abrupt change in the acoustic pressure distribution at the tissue surface, and consequently long simulation times, the background signal in the top three voxel planes was set to zero. This has a similar effect to the bandlimiting of the signal during measurement that would occur in practice and has no effect on the simulation of the artifacts around the vessels due to the limited detection aperture. Furthermore, in experimental images, the superficial layer is often stripped away to aid the visualization of the underlying structures. Similar approaches have been used to improve ${\mathrm{sO}}_{2}$ estimates generated by 2-D networks. In Ref. 22, the 10 most superficial pixel rows were removed from images before training to ensure that features deeper within the tissue (and therefore, dimmer than the comparatively bright superficial layers) were more detectable. Similarly, superficial voxel layers were removed from images in Ref. 21 to improve the accuracy of ${\mathrm{sO}}_{2}$ estimates.

Noise was added to each datapoint in the simulated pressure time series by adding a random number sampled from a Gaussian distribution with a standard deviation of 1% of the maximum value over all time series data generated from the same image, resulting in realistic SNRs of about 21 dB. Details of how this noise test was carried out and how the SNR was calculated are provided in Appendix A.

## 3 Network Architecture and Training Parameters

**Fig. 2:**
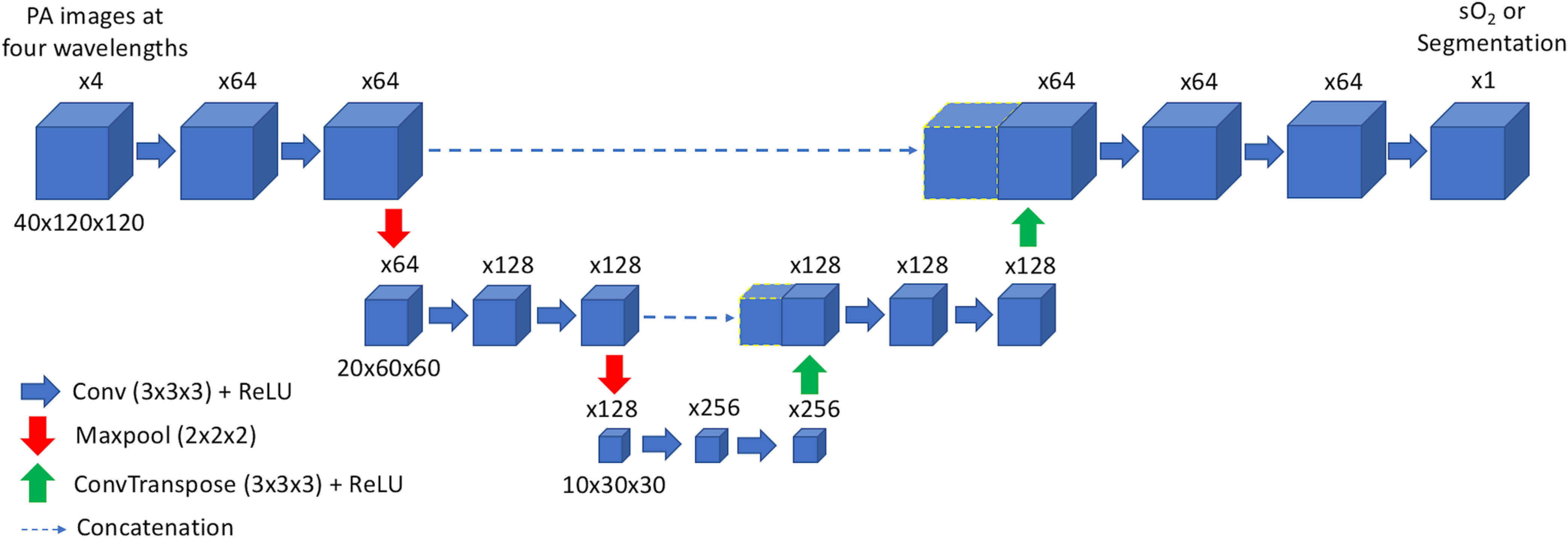
EDS network architecture. Blocks represent feature maps, where the number of feature maps generated by a convolutional layer is written above each block. Blue arrows denote convolutional layers, red arrows denote maxpooling layers, green arrows denote transposed convolutional layers, and dashed lines denote skip connections.

**Fig. 3:**
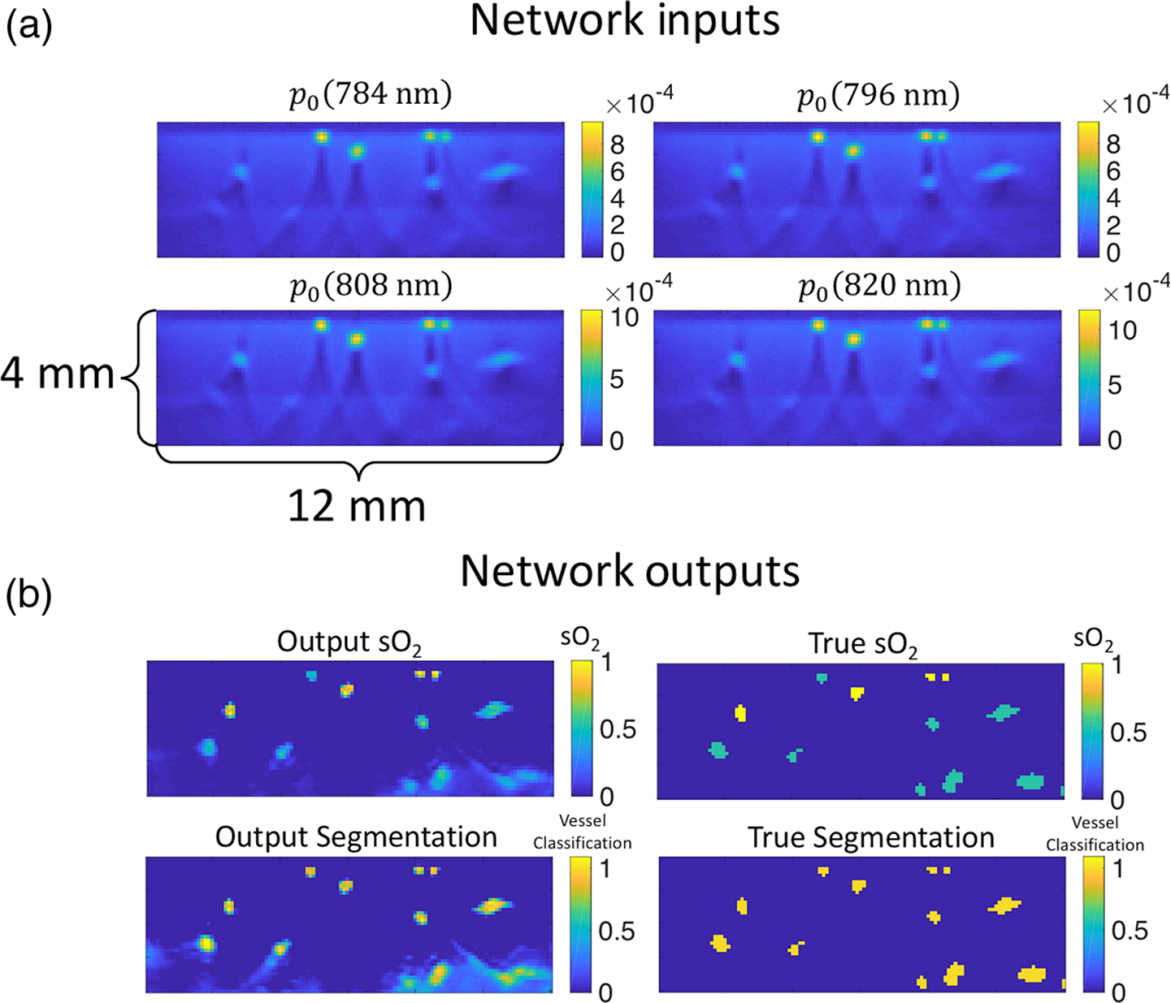
(a) 2-D slices of 3-D $p_{0}$ images simulated at four wavelengths from a single tissue model used as an input for networks A and B. (b) The corresponding 2-D slices of the 3-D outputs of the networks and the ground truths for this example.

A convolutional encoder–decoder type network with skip connections (EDS) (shown in Fig. 2 and denoted as network A) was trained to output an image of the ${\mathrm{sO}}_{2}$ distribution in each tissue model from 3-D image data acquired at four wavelengths. Another network, network B, was assigned an identical architecture to network A and was trained to output an image of vessel locations from the image sets (thereby segmenting the vessels). Figure 3 shows an example of the networks’ inputs and outputs. An EDS architecture was chosen for each task, as they have been shown to perform well at image-to-image regression tasks (i.e., tasks where the input data are a set of images and the output is an image).^44^ The architecture takes reconstructed 3-D images of a tissue model acquired at each excitation wavelength as an input. The multiscale nature of the network allows it to capture information about features at various resolutions and use image context at multiple scales.^45^^–^^47^ The network’s skip connections improve the stability of training and help retain information at finer resolutions. Finally, the network outputs a single 3-D feature map of the ${\mathrm{sO}}_{2}$ distribution or the vessel segmentation map.

An EDS network was trained to segment vessel locations because, although prior knowledge of the locations of vessels in the images was available for this *in silico* study, this information will not always be available when imaging tissues *in vivo*. Some technique for segmenting vessel positions from images is needed to enable the estimation of mean vessel ${\mathrm{sO}}_{2}$ values (the mean of the estimated ${\mathrm{sO}}_{2}$ values from all the voxels in a vessel) from the output map. Therefore, a vessel segmentation network was trained to show that neural networks can be used to acquire accurate mean vessel ${\mathrm{sO}}_{2}$ estimates without prior knowledge of vessel positions. The outputs of network B were only used to enable the calculation of mean vessel ${\mathrm{sO}}_{2}$ values without assumed prior knowledge of vessel positions and were not used to aid the training of network A. As will be discussed in Sec. 4, the output of the segmentation network also provides some information about where estimates in the output ${\mathrm{sO}}_{2}$ map may be more uncertain. This information can be used to improve mean vessel ${\mathrm{sO}}_{2}$ estimates by disregarding values from these regions. Two separate networks were trained for each task to limit additional bias in the learned features that would arise from training a single network to learn both tasks simultaneously. In Ref. 22, two different loss functions were used in a single network trained to produce both an image of the vascular ${\mathrm{sO}}_{2}$ distribution and an image of vessel locations. A different loss function was used for each task/branch of the network, where each function was arbitrarily assigned equal weights. Training two separate networks has the benefit that it removes the need to assign arbitrary weights to multiple loss functions that may be used to train a single network.

### 3.1 Training Parameters

**Fig. 4:**
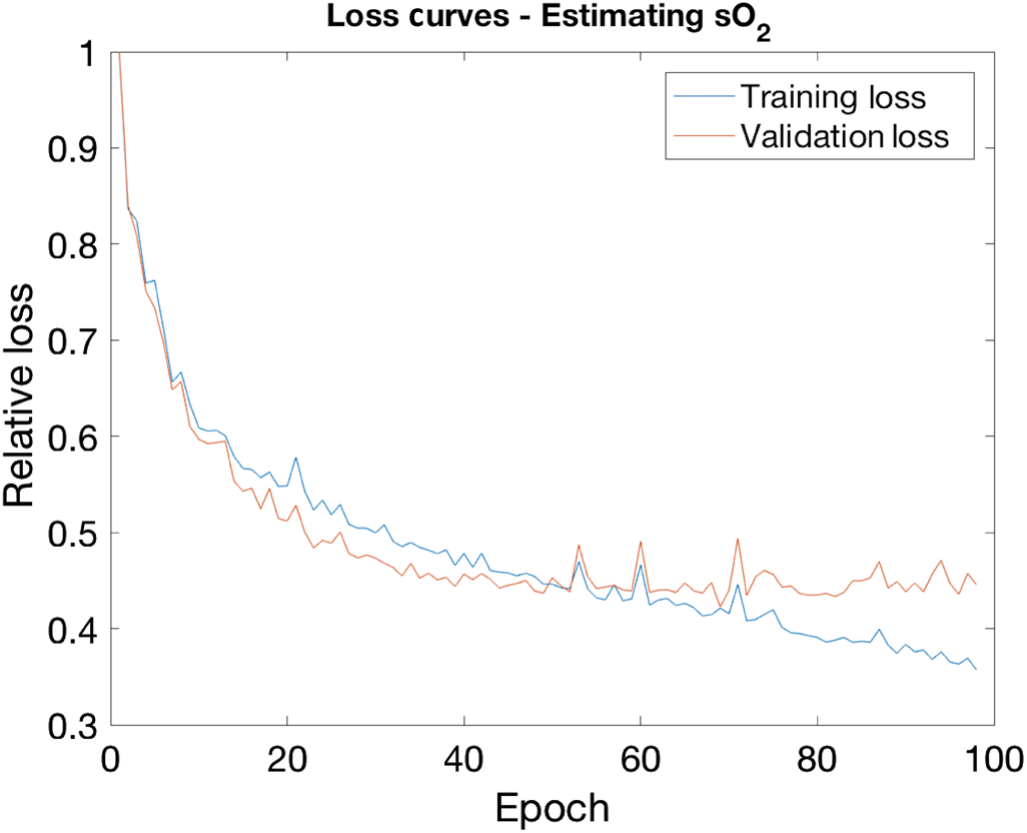
Relative loss curves ($\varepsilon_{A}$) for the ${\mathrm{sO}}_{2}$ estimating network.

Networks A and B were trained with 500 sets of images, corresponding to 500 different tissue models. An image of the true ${\mathrm{sO}}_{2}$ distribution of the vessels was used as the ground truth for network A. A binary image of true vessel locations was used as the ground truth for network B. Network A was trained for 98 epochs (loss curve shown in Fig. 4), while network B was trained for 84. Both networks were trained with a batch size of five image sets, a learning rate of $10^{-4}$, and with Adam as the optimizer. Training was terminated with an early stopping approach using a validation set of five examples. The networks were trained with the following error functionals, $\epsilon (\theta_{A})$ and $\epsilon (\theta_{B})$, (the norm of the squared difference between the network outputs and the ground truth images)

$$\epsilon_{\theta_{A}}={\left\|A_{\theta_{A}}[p_{0}(x,\lambda )]-{\mathrm{sO}}_{2}^{\text{true}}(x)\right\|}_{2}^{2},$$ (3)

and

$$\epsilon_{\theta_{B}}=\|B_{\theta_{B}}[p_{0}(x,\lambda )]-{\mathrm{seg}}^{\text{true}}(x){\|}_{2}^{2},$$ (4)

where $p_{0}(x,\lambda )$ are the multiwavelength images of each tissue model, ${\mathrm{sO}}_{2}^{\text{true}}(x)$ and ${\mathrm{seg}}^{\text{true}}(x)$ are the ground truth ${\mathrm{sO}}_{2}$ and vessel segmentation images, and $\theta_{A}$ and $\theta_{B}$ are the network parameters. Once trained, the networks $A_{\theta_{A}}$ and $B_{\theta_{B}}$ were evaluated on 40 test examples.

### 3.2 Output Processing

The mean ${\mathrm{sO}}_{2}$ of each vascular body was calculated using the voxels that had corresponding values ≥1 in the segmentation network (i.e., voxels that were confidently classified as belonging to a vessel by the segmentation network).

First, the indices associated with each major body in the segmentation network output [V(x) where x denotes the voxel index] were identified with the following method.

The output of the segmentation network V(x) (where x denotes the voxel index) was thresholded so all voxels with intensities <0.2 were set to zero, producing a new image $V^{\prime }(x)$. This was done to remove small values that connected all the vessels into one large body, ensuring each vessel was isolated in the volume. Then, the indices associated with each major body in $V^{\prime }(x)$ were identified using the bwlabeln() MATLAB function, generating a labeled image L(x), where all the voxels belonging to each independent connected body were assigned the same integer value, and each body in the image was assigned a unique value to be identified by (e.g., all the voxels belonging to a certain body were assigned a value of one, all the voxels in a different body were assigned a value of two, and so on).

Then, $V^{\prime }(x)$ was thresholded so all voxels with intensities <1 were set to zero, producing a new image $V^{\prime \prime }(x)$. This was done to isolate voxels where the network was confident that vessels were present. The output values from the segmentation network are approximately in the range 0 to 1 because the segmented training data images were binary, so the threshold of 0.2 (chosen empirically) was applicable to all the output images without requiring an additional normalization.

All the voxels in L(x) that now had values of zero in $V^{\prime \prime }(x)$ were also set to zero, producing a new image $L_{v}(x)$. Voxels that were once a part of the same body before this thresholding step may now be in separate bodies. However, their voxel ID retains information about which body they originally belonged to. This allows for the mean ${\mathrm{sO}}_{2}$ in each major vessel body to be calculated despite the thresholding (which removed voxels with low values in the segmentation network output) breaking up voxels that were once apart of the same body.

The mean ${\mathrm{sO}}_{2}$ of the voxels sharing the same integer value in $L_{v}(x)$ were calculated using the corresponding values in the output of the ${\mathrm{sO}}_{2}$ estimating network. The ground truth mean ${\mathrm{sO}}_{2}$ of the voxels sharing the same integer value in $L_{v}(x)$ were calculated using the values from the ground truth ${\mathrm{sO}}_{2}$ distribution.

## 4 Results and Discussion

**Fig. 5:**
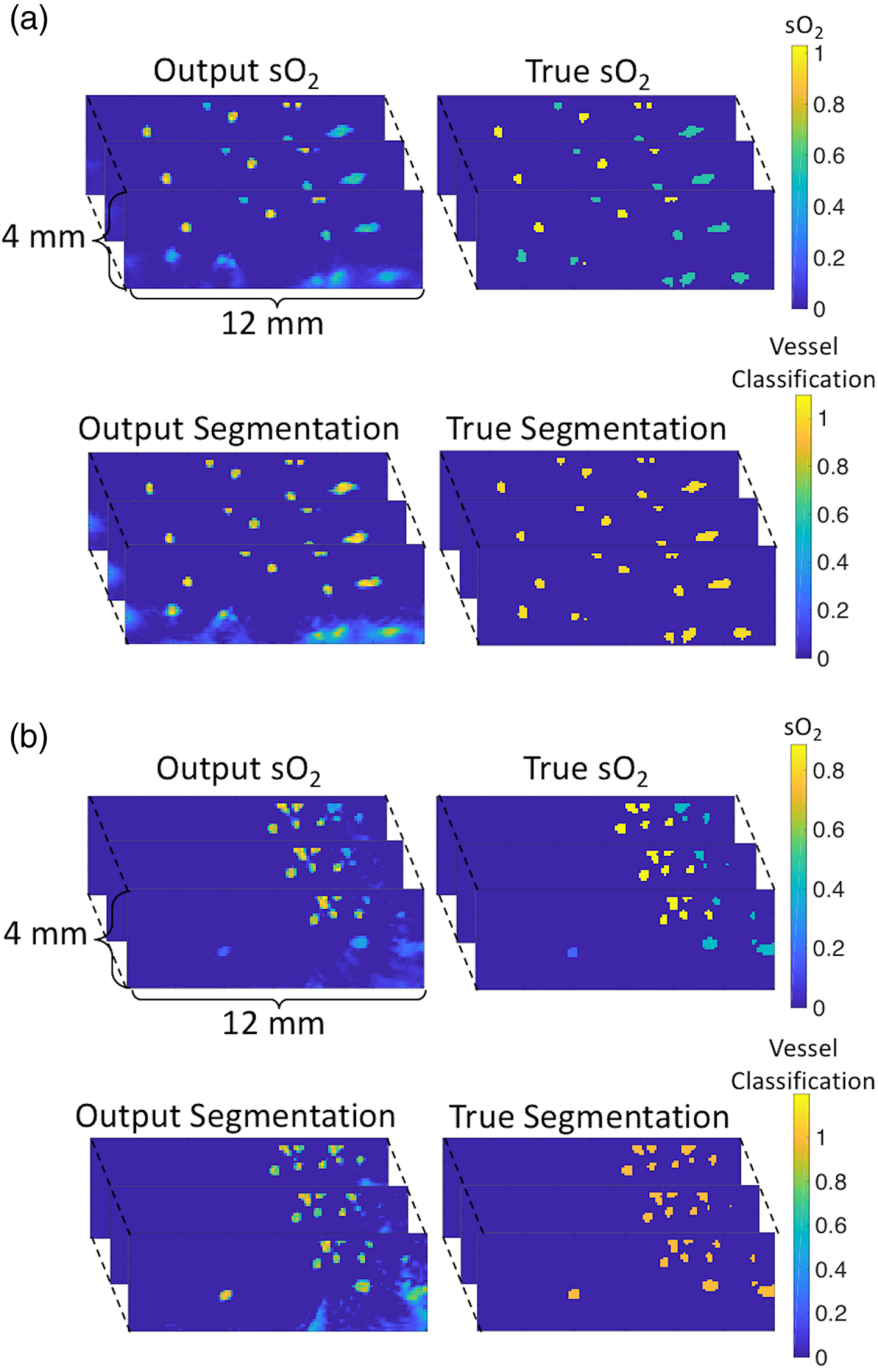
2-D slices of 3-D network outputs and corresponding ground truth ${\mathrm{sO}}_{2}$ and vessel segmentation images for two different tissue models (labeled a and b).

**Fig. 6:**
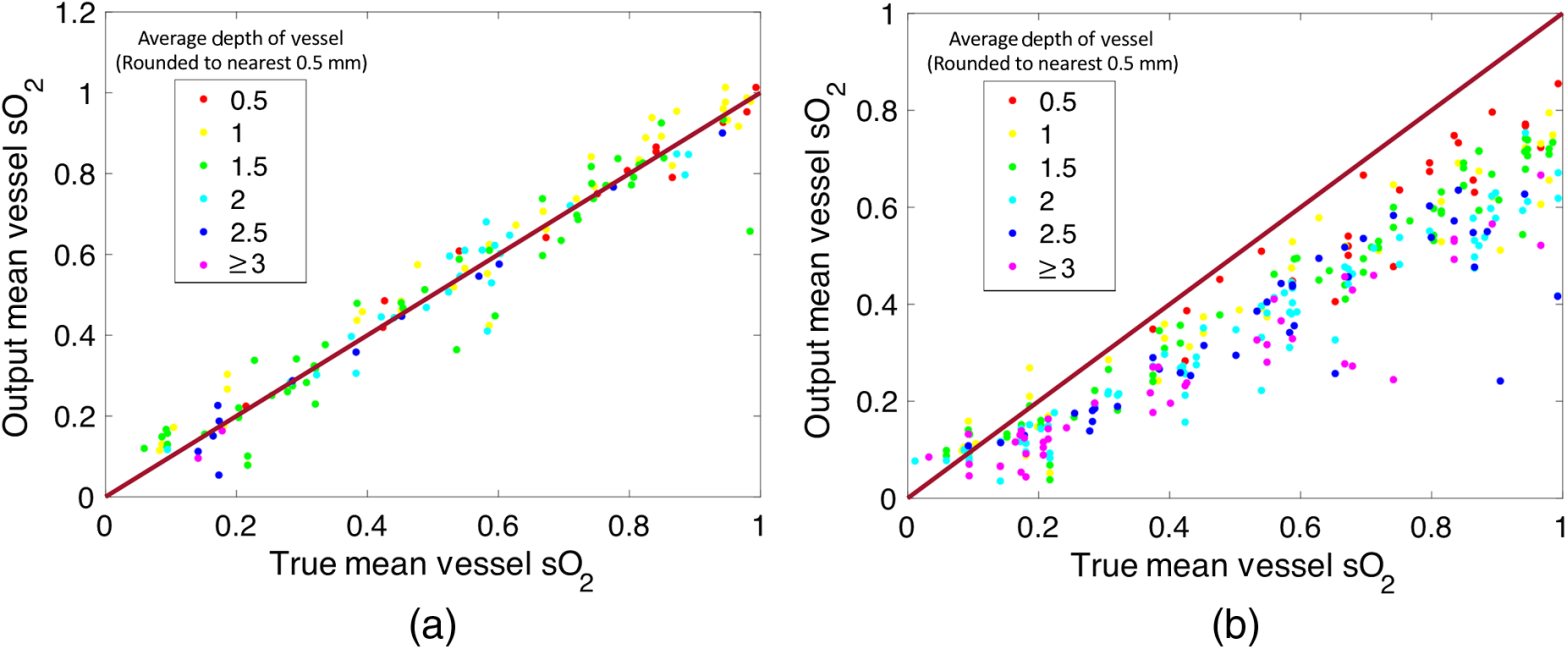
(a) Plot of the output mean vessel ${\mathrm{sO}}_{2}$ versus the true values for all the vessels in 40 tissue models not used for training, calculated with the voxels belonging to each vessel as determined by the segmentation network output. (b) Plot of the mean ${\mathrm{sO}}_{2}$ values for the same 40 tissue models calculated using the voxels known to belong to each vessel as determined by the ground truth vessel positions. These plots show that using the output of the segmentation network in combination with the output of the ${\mathrm{sO}}_{2}$-estimating network significantly improves the accuracy of the estimates.

The 3-D image outputs of both the ${\mathrm{sO}}_{2}$-estimating and segmentation networks were processed in order to calculate the mean ${\mathrm{sO}}_{2}$ in each major vessel body using only the ${\mathrm{sO}}_{2}$ estimates from voxels that the segmentation network was confident contained vessels (the reason for using the segmentation output was so that the mean vessel ${\mathrm{sO}}_{2}$ could be calculated without *a priori* knowledge of vessel locations that might not be available in an *in vivo* scenario). More details about how this process was performed are provided in Sec. 3.2. The mean of the absolute difference between the true mean vessel ${\mathrm{sO}}_{2}$ and the output mean vessel ${\mathrm{sO}}_{2}$ over all 40 sets of images was 4.4%, and the standard deviation of the absolute difference between the true mean vessel ${\mathrm{sO}}_{2}$ and the output mean vessel ${\mathrm{sO}}_{2}$ was 4.5% (some 2-D image slices taken from the networks’ 3-D outputs are shown in Fig. 5, and a plot of all the estimates is provided in Fig. 6). Therefore, on average, the predicted mean vessel ${\mathrm{sO}}_{2}$ was within 5% of the true value. The mean difference between the true mean vessel ${\mathrm{sO}}_{2}$ and the output mean vessel ${\mathrm{sO}}_{2}$ was −0.3% with a standard deviation of 6.3%. The typical error for a mean vessel ${\mathrm{sO}}_{2}$ estimate was thus between −6.6% and 6.0%.

To assess the effect that using the output of the segmentation network may have had on the accuracy of the ${\mathrm{sO}}_{2}$ estimates, the mean ${\mathrm{sO}}_{2}$ of each vascular body in the network output was estimated using the voxels known to belong to each vessel, as opposed to the voxels assigned to each body by the segmentation network output. Curiously, the accuracy of the estimates decreased when the ground truth vessel voxels were used for calculating mean ${\mathrm{sO}}_{2}$ values. Figure 6 shows a plot of the results over 40 tissue models. The mean of the absolute value of the offset between the true value and the network output was 16.6%, the mean offset was 16.2%, and the standard deviation of the offset was 11.5%. This suggests that regions where the segmentation network confidently classified as belonging to a vessel corresponded to regions where the ${\mathrm{sO}}_{2}$ network was more accurate. Furthermore, it is clear that the accuracy of the ${\mathrm{sO}}_{2}$ estimates calculated using the ground truth vessels positions decreases with depth. We do not observe this in the estimates calculated using the output of the segmentation network, suggesting that the use of the segmentation network corrects for the depth dependence of the accuracy of network A.

Even though both networks are trained separately, they share the same input data, network architecture, and are trained with the same loss function where only the distribution of values in the corresponding ground-truth varies (continuous versus binary). As such, it is not surprising that the learned mapping properties are similar and complement each other. The L2 loss was used for training network B (as opposed to a binary classification loss function that would normally be used for a segmentation task) to ensure that the network outputs would retain more information about the uncertainty of estimates.

Both networks A and B reflect the limited-view nature of the data in their outputs, hence the positions of the vessels in both differ similarly from the ground truth. The accuracy of the output of both networks decreased with the depth of the vessels, i.e., the distance from the detector array, as can be seen in Fig. 5. There are a couple of reasons for why this might be the case. First, image SNR decreases with depth. The image SNR decreases with depth both because the fluence decays with depth and because of the depth-dependence of the limited-view reconstruction artifacts. Second, these artifacts become more spread in out in space with depth, introducing greater uncertainty as to the shape and location of the vessels. The output of both networks is least accurate in the deepest corners of each image, where the artifacts are the most significant.

Filters in a convolutional layer are the same wherever they are applied in the image, they are spatially invariant, and are, therefore, most suited to detecting features that are also spatially invariant. However, the limited-view artifacts are not; they are small close to the center of the sensor array and become more significant further away. The multiscale nature increases the receptive field and hence locality can be learned by the network. Nevertheless, we decided to limit the receptive field using a slightly smaller network architecture than the classic U-Net. In this way, we retain uncertainty in the deeper tissue layers instead of introducing a learned bias.

The 3-D results shown in Figs. 5 and 6 are of comparable accuracy to results from other groups obtained by training 2-D convolutional neural networks to process 2-D images (lacking the presence of reconstruction artifacts) of simpler 2-D tissue models.^22^^,^^24^^,^^25^ The technique presented here was not only able to handle more complex tissue models (the tissue models presented here feature more realistic vascular architectures and multiple skin layers with varying thicknesses and optical properties), but also took as the input data 3-D images featuring noise and reconstruction artifacts. Unlike networks trained on 2-D images sliced from 3-D images of tissue models (such as those used in Ref. 21), the 3-D networks were able to use information from entire 3-D image volumes to generate estimates. Because the fluence distribution and limited-view artifacts are 3-D in nature, learning 3-D features is more efficient than trying to learn to represent 2-D sections/slices through 3-D objects with 2-D feature maps. This likely increases their ability to produce accurate estimates in more complex tissue models. Despite being more sophisticated than other tissue models used to date, the tissue models used here were nevertheless created with some simplifying assumptions. Each skin layer was assigned a planar geometry, where the value of the optical properties associated with each layer at each wavelength remained constant within each layer (e.g., the scattering coefficient of the epidermis was constant within the epidermis layer). Although the absorption coefficient of each layer was varied for each tissue model, the scattering coefficient of each skin type remained constant (but did vary with wavelength). Other experimental factors that can affect image amplitude, such as the directivity of the acoustic sensors, were not incorporated into the simulation pipeline. It remains to be seen the extent to which these assumptions will hold true when this network is applied to *in vivo* data. To ensure that networks initially trained on simplified simulated images can output accurate estimates when provided real images, networks may have to be modified with transfer training, taking advantage of datasets of real images.^36^^,^^48^^,^^49^ Looking beyond the complexity of the tissue models, there are other more fundamental challenges that will make the application to living tissue nontrivial. In order to train a network using a supervised learning approach with *in vivo* data (or even to validate any technique for estimating ${\mathrm{sO}}_{2}$ *in vivo*), the corresponding ground truth ${\mathrm{sO}}_{2}$ distribution must be available. It is unclear as to how this information might be acquired, and this poses a significant challenge that must be overcome to realize or validate the application of the technique. As an intermediate step toward generating *in vivo* datasets, blood flow phantoms with tuneable ${\mathrm{sO}}_{2}$ could be used to generate labeled data in conditions mimicking realistic imaging scenarios.^33^^,^^34^ Although it is important to show that a network can cope with all the confounding effects present in real images of tissue, it is still interesting and important to know that the technique can cope with at least some of the challenges faced in such scenarios. This work provides an essential demonstration of the technique’s ability to generate accurate 3-D estimates from 3-D image data despite the presence of some confounding experimental effects that distort image amplitude, and despite some variation in the distribution of tissue types and the distribution of vessels for each tissue model.

## 5 Conclusions

Data-driven approaches have been shown capable of recovering sample optical properties and maps of ${\mathrm{sO}}_{2}$ from 2-D PA images of fairly simple tissue models. However, because the fluence distribution and limited-view artifacts are 3-D, 2-D networks are at a disadvantage as they must learn to represent 2-D sections/slices through 3-D objects with 2-D feature maps. Networks that can process whole 3-D images with 3-D filters are more efficient as they can detect 3-D features, and this likely increases their ability to produce accurate estimates in more complex tissue models. There may be cases where accurate ${\mathrm{sO}}_{2}$ maps may only be generated with 3-D network architectures. Therefore, to assess whether data-driven techniques have the potential to provide accurate estimates in realistic imaging scenarios, it is essential to demonstrate a neural network’s ability to process 3-D image data to generate ${\mathrm{sO}}_{2}$ estimates. The capability of an EDS to generate accurate maps of vessel ${\mathrm{sO}}_{2}$ and vessel locations from multiwavelength simulated images (containing noise and limited view artifacts) of tissue models featuring optically heterogeneous backgrounds (with varying absorption properties) and realistic vessel architectures was demonstrated. Regions where the segmentation output was confident in its predictions of vessel locations corresponded to more accurate regions in the ${\mathrm{sO}}_{2}$-estimating network output. As a consequence, the accuracy of the network’s mean vessel ${\mathrm{sO}}_{2}$ estimates improved when the output of the segmentation network was used to determine vessel locations as opposed to the ground truth. In contrast to both analytical and iterative error-minimization techniques, the networks were able to generate these estimates without total knowledge of each tissues’ constituent chromophores, or an accurate image generation model—both of which would not normally be available in a typical *in vivo* imaging scenario. This work shows that fully convolutional neural networks can process whole 3-D images of tissues to generate accurate 3-D images of vascular ${\mathrm{sO}}_{2}$ distributions, and that accurate estimates can be generated despite some degree of variation in the distribution of tissue types, vessels, and the presence of noise and reconstruction artifacts in the data.

## 6 Appendix A: Noise Test

Noise was incorporated into the simulated images by adding it to the simulated pressure time series before the reconstruction step. Noise was added to each datapoint in the simulated pressure time series by adding a random number sampled from a Gaussian distribution with a standard deviation of 1% of the maximum value over all time series data generated from the same image, resulting in realistic SNRs of 20.9, 21.3, 21.4, and 21.4 dB for a set of images of a single tissue model simulated at 784, 796, 808, and 820 nm, respectively. The details of this measurement are described in the following section.

1. A single tissue model was defined.
2. A fluence simulation was run 20 times (each run indexed with r) for each excitation wavelength (λ) with $10^{9}$ photons to produce $\Phi_{r}(x,\lambda )$, where x indices the voxels in the simulation output. The optical properties of the tissue model at each excitation wavelength were identical for all 20 runs.
3. A set of initial pressure distributions, $I_{r}(x,\lambda )$, were generated from the fluence simulations
  $$I_{r}(x,\lambda )=\Phi_{r}(x,\lambda )\mu_{a}(x,\lambda )\Gamma .$$ (5)
4. The emission and detection of pressure time series were simulated in k-Wave to generate simulated pressure time series $p_{r,j}(t,\lambda )$, where j indexes each time series produced by the simulation, and t is the simulation time (simulation parameters were identical to those outlined in Sec. 2.3).
5. Some amount of noise $n_{r,j}(t,\lambda )$ was added to each point in each pressure time series
  p^r,j(t,λ)=nr,j(t,λ)+pr,j(t,λ), (6)
  where $n_{r,j}(t,\lambda )$ was determined by sampling a random value from a Gaussian distribution with a standard deviation of $cn_{\max}(\lambda )$, where $n_{\max}(\lambda )$ is the max value of $p_{r,j}(t,\lambda )$ over all j and t for a given λ and r (i.e., the max value of all the time series for a given run at a given wavelength), while c is the proportion of this value used to define the standard deviation.
6. The images were reconstructed in k-Wave with time reversal to produce $I_{r}^{\mathrm{Recon}}(x,\lambda )$.
7. The mean and standard deviation for each voxel for each wavelength over all 20 runs was calculated with
  $$\mu (x,\lambda )=\frac{1}{20}\overset{20}{\underset{r=1}{\sum }}I_{r}^{\mathrm{Recon}}(x,\lambda ),$$ (7)
  and
  $${\sigma (x,\lambda )=\{\frac{1}{19}\overset{20}{\underset{r=1}{\sum }}{\left[I_{r}^{\text{Recon}}(x,\lambda )-\mu (x,\lambda )\right]}^{2}\}}^{\frac{1}{2}}.$$ (8)
8. The SNR of each voxel at each wavelength was calculated with
  $$\mathrm{SNR}(x,\lambda )=20\;\log_{10}[\frac{\mu (x,\lambda )}{\sigma (x,\lambda )}].$$ (9)
9. For each wavelength, the mean of the SNR values over all voxels V was calculated with
  $$\mu_{\mathrm{SNR}}(\lambda )=\sum_{x=1}^{V}\mathrm{SNR}(x,\lambda ).$$ (10)

Because the SNR depends on the optical properties of objects in the sample domain, the SNR will vary depending on the tissue model used for the test. Here, we only use a single tissue model with a single set of tissue properties to obtain some approximate idea of how much noise features in the simulated images.

## 7 Appendix B: Optical Properties of Skin Layers

The refractive index, anisotropy factor, optical absorption coefficient, and optical scattering coefficient of each tissue or chromophore are required to construct a tissue model for a MCXLAB simulation. Here, we tabulate expressions for computing the relevant quantities or list the values of certain quantities for various wavelengths in Table 1. These values/resources were chosen as they featured data in the wavelength range for our simulations.

**Table 1:** Skin optical properties (λ is given in nm).

Tissue	Parameter	Value	Ref.
Epidermis	Optical absorption (${\mathrm{cm}}^{-1}$)	$\mu_{\mathrm{ae}}=[C_{M}6.6(\lambda^{-3.33})(10^{11})]+(1-C_{M})\{0.244+85.3[\exp(-\frac{\lambda -154}{66.2})]\}$	50
	Melanosome fraction $C_{M}$	6% for Caucasian skin, 40% for pigmented skin	51
	Reduced scattering (${\mathrm{cm}}^{-1}$)	$\mu_{s}^{\prime }=68.7(\frac{\lambda }{500}{)}^{-1.16}$	52
	Refractive index	1.42–1.44 (700 to 900 nm)	53
	Anisotropy	0.95–0.8 (700 to 1500 nm)	54,60
	Thickness	0.1 mm	
Dermis	Optical absorption (${\mathrm{cm}}^{-1}$)	$\mu_{\mathrm{ad}}=C_{B}\mu_{\mathrm{ab}}+(1-C_{B})\{0.244+85.3[\exp(-\frac{\lambda -154}{66.2})]\}$	50
	Blood volume fraction $C_{B}$	0.2% to 7%	55
	Reduced scattering (${\mathrm{cm}}^{-1}$)	$\mu_{s}^{\prime }=45.3{\left(\frac{\lambda }{500}\right)}^{-1.292}$	52
	Refractive index	$n=A+\frac{B}{\lambda^{2}}+\frac{C}{\lambda^{4}}$, where A=1.3696, $B=3.9168\times 10^{3}$, $C=2.5588x10^{3}$	53
	Anisotropy	0.95 – 0.8 (700 to 1500 nm)	54,60
	${\mathrm{sO}}_{2}$	40% to 100%	55
Blood	Optical absorption (${\mathrm{cm}}^{-1}$)	$\mu_{\mathrm{ab}}=C_{\mathrm{Hb}}\alpha_{\mathrm{Hb}}+C_{{\mathrm{HbO}}_{2}}\alpha_{{\mathrm{HbO}}_{2}}$	50
	Reduced scattering (${\mathrm{cm}}^{-1}$)	$22{\left(\frac{\lambda }{500}\right)}^{-0.66}$	52
	Refractive Index	1.36 (680 to 930 nm)	56
	Anisotropy	0.994 (roughly constant for variant wavelength and ${\mathrm{sO}}_{2}$)	57,58
Hypodermis	Optical absorption (${\mathrm{cm}}^{-1}$)	1.1 at 770 nm, 1.0 at 830 nm	59
	Reduced scattering (${\mathrm{cm}}^{-1}$)	20.7 at 770 nm, 19.6 at 830 nm	59
	Refractive index	1.44 (456 to 1064 nm)	54
	Anisotropy	0.8 (700 to 1500 nm)	60
